# Transmission of molecularly undetectable circulating parasite clones leads to high infection complexity in mosquitoes post feeding^☆^

**DOI:** 10.1016/j.ijpara.2018.02.005

**Published:** 2018-07

**Authors:** Lynn Grignard, Bronner P. Gonçalves, Angela M. Early, Rachel F. Daniels, Alfred B. Tiono, Wamdaogo M. Guelbéogo, Alphonse Ouédraogo, Elke M. van Veen, Kjerstin Lanke, Amidou Diarra, Issa Nebie, Sodiomon B. Sirima, Geoff A. Targett, Sarah K. Volkman, Daniel E. Neafsey, Dyann F. Wirth, Teun Bousema, Chris Drakeley

**Affiliations:** a Department of Immunology and Infection, London School of Hygiene & Tropical Medicine, London, UK; b Broad Institute of MIT and Harvard, Cambridge, MA, USA; c Department of Immunology and Infectious Disease, Harvard T.H. Chan School of Public Health, Boston, MA, USA; d Department of Biomedical Sciences, Centre National de Recherche et de Formation sur le Paludisme, Ouagadougou, Burkina Faso; e School of Nursing and Health Sciences, Simmons College, Boston, MA, USA; f Radboud Institute for Health Sciences, Radboud University Medical Center, Nijmegen, The Netherlands

**Keywords:** Malaria, Transmission, Multiplicity of infection, Mosquito

## Abstract

*Plasmodium falciparum* malaria infections often comprise multiple distinct parasite clones. Few datasets have directly assessed infection complexity in humans and mosquitoes they infect. Examining parasites using molecular tools may provide insights into the selective transmissibility of isolates. Using capillary electrophoresis genotyping and next generation amplicon sequencing, we analysed complexity of parasite infections in human blood and in the midguts of mosquitoes that became infected in membrane feeding experiments using the same blood material in two West African settings. Median numbers of clones in humans and mosquitoes were higher in samples from Burkina Faso (4.5, interquartile range 2–8 for humans; and 2, interquartile range 1–3 for mosquitoes) than in The Gambia (2, interquartile range 1–3 and 1, interquartile range 1–3, for humans and mosquitoes, respectively). Whilst the median number of clones was commonly higher in human blood samples, not all transmitted alleles were detectable in the human peripheral blood. In both study sample sets, additional parasite alleles were identified in mosquitoes compared with the matched human samples (10–88.9% of all clones/feeding assay, *n* = 73 feeding assays). The results are likely due to preferential amplification of the most abundant clones in peripheral blood but confirm the presence of low density clones that produce transmissible sexual stage parasites.

## Highlights

• Additional parasite alleles were consistently identified in mosquitoes compared with the human blood sample they had fed on.
• Assessments of *Plasmodium falciparum* complexity relying on single time-point collections miss transmissible clones.
• Low-density gametocyte – producing clones are capable of successfully establishing infections in mosquitoes.

## Graphical abstract

**Figure:**
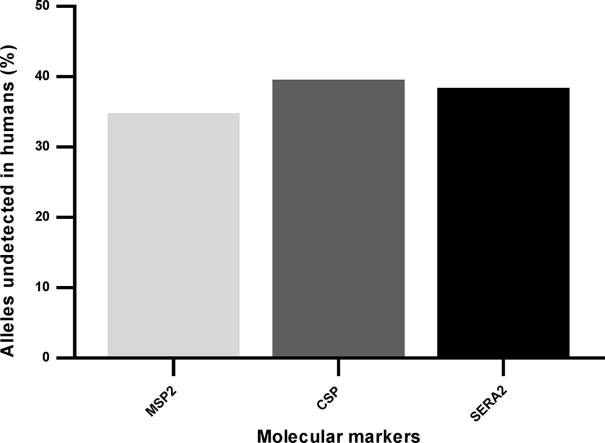

## 1 Introduction

The transmission of malaria from human to mosquito starts with the development of gametocytes from their asexual progenitors. These haploid male and female gametocytes are ingested by a mosquito during feeding and fuse to form a diploid zygote. This zygote develops into a motile ookinete that traverses the midgut wall and develops into an oocyst which after 10–14 days ruptures to release sporozoites that render the mosquito infectious (Aly et al., 2009, Sinden, 2015). Factors influencing transmission success include transmission intensity, seasonality, human exposure and attractiveness to mosquitoes, immune responses in both humans and mosquitoes, gametocyte fitness and densities; and multiplicity/complexity of infection (Bousema and Drakeley, 2011, Wampfler et al., 2014, Nilsson et al., 2015, Stone et al., 2015). Multiplicity of infection (MOI) denotes the number of clones within an infection without information as to kind or type. Complexity of infection (COI) reflects not just a number, but more information by providing a haplotype or other genetic signature for those parasite types.

In most endemic settings, malaria infections are commonly polyclonal either as a consequence of multiple infectious bites or of mosquito bites with several clones (Juliano et al., 2010). Since all naturally circulating falciparum malaria parasites are thought to produce gametocytes, this implies that these multiple parasite clones (Wampfler et al., 2014) all contribute to transmission to mosquitoes, although their infectivity is likely to differ: clones established earlier during infections are more likely to have gametocytes that completed their long maturation process, and clones that are present at higher asexual densities might also have higher sexual stage densities. The relative transmissibility of distinct clones in polyclonal infections and their contribution to transmission remains largely unexplored. This has relevance for drug and vaccine studies, and whether clones that are resistant are also more transmissible (Cattamanchi et al., 2003, Mharakurwa et al., 2011, Mharakurwa et al., 2013, Mendes et al., 2013, Neafsey et al., 2015).

Current methods to assess the number of clones in an infection test for allelic size polymorphisms either by conventional or capillary electrophoresis (CE) genotyping of markers such as merozoite surface proteins 1 and 2 (MSP1, MSP2) and glutamate-rich protein (GLURP) (Cattamanchi et al., 2003, Liljander et al., 2009, Schoepflin et al., 2009, Mwingira et al., 2011). Other, sequence based, assays include: PCR/RFLP, microsatellite genotyping (Escalante et al., 2015), single nucleotide polymorphism (SNP) panel barcodes that uniquely identify and track parasites in an infection (Daniels et al., 2008) and deep sequencing (Juliano et al., 2010, Assefa et al., 2014). There a number of limitations in these different assays: conventional electrophoresis is not as sensitive as CE (Schoepflin et al., 2009) and all DNA-based assays fail to distinguish between asexual parasites and gametocytes. Gametocyte RNA markers investigated to date are less diverse compared with DNA markers and include Pfg377 and Pfs230 (Nwakanma et al., 2008, Wampfler et al., 2014). Sensitivity of the CE-based genotyping methods depends on highly diverse markers. MSP1 and MSP2 are superior to GLURP (Falk et al., 2006). Barcode techniques and deep sequencing are very sensitive but some require highly concentrated, high quality DNA and are computationally complicated for polygenomic infections (Daniels et al., 2008). Two recent studies used gametocyte RNA-based typing or microsatellite typing to assess both human and mosquito clonal diversity in *Plasmodium falciparum* (Nwakanma et al., 2008, Morlais et al., 2015) and reported that monoclonal infections lead to higher infection prevalence and oocyst burden compared with more complex infections.

In this study, we investigate the MOI and the clonal diversity in human hosts and in mosquitoes that become infected during feeding experiments from two different endemic settings using two different genotyping methods. Whole blood samples comprising asexual and sexual parasites were assayed, as were oocysts representing clones successfully transmitted to mosquitoes. Magnetic gametocyte enrichment was used to identify clones more likely to be transmitted. We observed that in both The Gambia and Burkina Faso, a considerable fraction of infectious clones was detectable in mosquito oocysts but not in the peripheral human blood.

## 2 Materials and methods

### 2.1 In vivo transmission studies

Human blood samples infected with *P. falciparum* and mosquitoes infected by direct membrane feeding assay (DMFA) were obtained from studies performed in The Gambia and Burkina Faso. In Burkina Faso, samples were prospectively collected with the specific aim to understand the COIs in humans and mosquitoes, and sample collection from human donors was designed to maximise the resolution in identifying all transmissible parasite clones (see Section 2.3). The analyses were supplemented with archived samples collected during previous studies in The Gambia where we hypothesised a lower COI and thus, potentially, a better ability to genotype the majority of circulating parasites. At both sites asexual stage and gametocyte parasite counts were based on microscopic examination of 100 high power fields (Supplementary Table S1).

Samples obtained in The Gambia were from two previously described clinical studies conducted at the Medical Research Council (MRC) field station in Farafenni. In the first study, in 1994, individuals with gametocytes were recruited on presentation or 7 days later after treatment and asked to provide blood for mosquito feeding assays (Drakeley et al., 1999). In 1999, children were recruited at the government health centres in Farafenni town and enrolled in a study aiming to assess infectivity after malaria (Targett et al., 2001). From this study, blood samples were collected 7 days after treatment with either pyrimethamine/sulfadoxine (PSD) alone or with PSD plus three doses of artesunate (AS).

Samples from Burkina Faso were collected in Laye (2013) and Balonghin (2014), two villages located in the north-west and in the south of the capital, Ouagadougou. A total of 400 individuals were recruited regardless of their parasite status and invited to attend the Centre National de la Recherche et de la Formation sur le Paludisme (CNRFP) in Ouagadougou for feeding experiments. (Gonçalves et al., 2017). Additional samples (*n* = 38) were included from feeds on microscopy-positive gametocyte carriers who were recruited alongside community surveys as quality controls for the performance of mosquito feeding experiments. Written informed consent was obtained from all study participants or their guardians.

Ethical clearance in Burkina Faso was approved by the ethical review committees of the Ministry of Health of Burkina Faso and of the London School of Hygiene and Tropical Medicine, UK (reference number 6271). The Gambia 1994 and 1999 studies were approved by the Medical Research Council/Gambia Government Joint Ethical Committee. In The Gambia, unique reference numbers were not provided with ethical approval at the time.

### 2.2 Membrane feeding of mosquitoes

The mosquito feeds were performed as described elsewhere (Drakeley et al., 1999, Targett et al., 2001, Bousema et al., 2012). Briefly, in The Gambia, 3–5 day old F1 progeny of locally caught *Anopheles gambiae* sensu stricto (ss) were used in the feeding assays. In Burkina Faso, laboratory strains of *An. gambiae* were used. Whole blood (400–1000 µl) was added to glass feeders pre-warmed to 37 °C and mosquitoes were allowed to feed for 15–20 min. Fully fed mosquitoes were retained and mosquitoes were dissected on day 7 after the feed. Oocyst-positive midguts were suspended in 1 ml of 70% ethanol (The Gambia) or 50 µl of RNA protect (Qiagen, UK) Burkina Faso) and stored at −80 °C until processing.

### 2.3 Magnetic enrichment of gametocytes

In a subset of experiments from Burkina Faso, the parasite population was enriched for gametocytes prior to molecular parasite detection. This was performed using MACS LS columns (Miltenyi Biotec, UK) as previously published (Karl et al., 2011) with minor modifications. Immediately after venipuncture, 500 µl of whole blood were re-suspended in 5 ml of magnetic fractionation buffer (1× PBS pH7.4, 0.5% BSA, 2 mM EDTA) and passed in batches over the LS column. The flow-through, containing asexual parasites, was collected and briefly centrifuged, and the supernatant was removed and the cell pellet re-suspended in 500 µl of RNA protect (Qiagen). The gametocyte fraction was eluted, briefly centrifuged and suspended in 5× RNA protect (Qiagen). All steps were performed at 37 °C.

### 2.4 DNA extraction

#### 2.4.1 Mosquito midguts

Oocyst DNA was extracted using the phenol/chloroform method and eluted in 20 µl of nuclease-free water (Ranford-Cartwright et al., 1993).

#### 2.4.2 Human samples

Human blood samples (100 µl) from The Gambia (both years) were extracted using the Boom method (Boom et al., 1990) and eluted in 50 µl of nuclease-free water (Ambion, UK). One hundred microliters of whole blood samples from the Burkina Faso studies were processed using the MagNAPure Total Nucleic Acid Isolation Kit – High Performance (Roche, The Netherlands) and eluted in 50 µl of nuclease-free water. Asexual parasite samples were extracted using the MagNAPure LC DNA Isolation Kit – Large Volume (Roche) and eluted in 100 µl of nuclease-free water. Enriched gametocyte DNA was extracted by an AllPrep DNA/RNA Micro kit (Qiagen) and eluted in 100 µl of elution buffer according to the manufacturer’s guide.

### 2.5 Genotyping of human blood samples and mosquito oocysts

#### 2.5.1 MSP2 CE genotyping

For the MSP2 CE genotyping, 5 µl of extracted DNA were amplified in a nested PCR specific for MSP2 3D7 and Fc27 allelic families (Falk et al., 2006, Schoepflin et al., 2009). Diluted PCR product, 2.5 µl per sample, was processed on an ABI 7500 fast (Applied Biosystems, UK) at the Medical Research Council Genomics Core Facility (Imperial College, Hammersmith Hospital Campus, UK). The data were analysed using GeneMapper software version 4.0 (Applied Biosystems). The different MSP2 alleles were sized and averaged to create 3 bp bins.

#### 2.5.2 Amplicon sequencing

The amplicon sequencing was performed as previously described (Neafsey et al., 2015). The assay targets circumsporozoite protein (CSP) and serine repeat antigen 2 (SERA2). In brief, an approximate 300 nucleotide (nt) region of each gene was amplified with PCR and then sequenced using overlapping 250 bp paired-end miSeq Illumina reads. After sequencing, reads with the same barcode were joined and used to call raw haplotypes. Haplotypes from within each sample were clustered. If any haplotypes were separated by a single nt change and there was an eight-fold or greater difference in their abundance, the less prevalent haplotype was discarded. Further, within each sample, haplotypes were discarded if those were represented by less than five read pairs or less than 5% of the total reads for a sample. The data have been deposited with links to BioProject accession number PRJNA451490 in the National Center for Biotechnology Information (NCBI) BioProject database (https://www.ncbi.nlm.nih.gov/bioproject/).

### 2.6 Statistical analysis

All data were analysed using STATA Version 13 (Statacorp, Texas, USA) and GraphPadPrism Version 6 (GraphPad Software Inc., La Jolla, USA). Allelic richness and allele frequencies were estimated using FSTAT (FSTAT, version 293, Jérôme Goudet, Université de Lausanne, Switzerland). Non-parametric tests were used to analyse the relationship between number of clones and surveys, age and parasite densities. *P* < 0.05 was considered significant.

## 3 Results

### 3.1 Molecular infection characteristics in humans

#### 3.1.1 Multiplicity of infection in humans (MOI_H_)

A total of 307 human blood samples were analysed by MSP2 CE genotyping, 81 from The Gambia and 226 from Burkina Faso. Overall, 24.8% (76/307) of infections were monoclonal and 75.2% (231/307) were polyclonal. The proportion of monoclonal infections was higher in The Gambia (48%, 39/81) than in Burkina Faso (16.4%, 37/226). The median number of clones per positive human sample (MOI_H_) for each study is shown in Table 1. MOI_H_ was highest in Burkina Faso during the dry season and lowest in The Gambia 1994 study, most likely related to transmission intensity which is higher in Burkina Faso. There was no difference in the median number of clones between the wet and dry seasons in Burkina Faso (Table 1). Participants from Burkina Faso were stratified into three age groups: <5 years old, 5–15 years old and >15 years old. Age stratification of the data (Supplementary Table S2) showed that individuals less than 15 years of age had significantly more clones than children under 5 years old (Mann–Whitney, *P* = 0.04) and the adults (Mann–Whitney, *P* = 0.01). In The Gambia only two MSP2-positive samples were from adults, hence MOI_H_ was not stratified by age. Infections in The Gambia 1999 samples, which were all collected 7 days after drug treatment, still contained similarly diverse parasites (MOI_H_ = 2); no difference in MOI_H_ in between treatment arms was detected (although the study was not designed to detect this) (Supplementary Table S3).

**Table 1:** Median number of *Plasmodium falciparum* clones and allelic richness in all human blood samples, infectious individuals and in mosquitoes, and interquartile range (MOIH/M, IQR) by study.

	The Gambia 1994			The Gambia 1999			Burkina Faso (dry season)			Burkina Faso (wet season)		
	All	Infectious	Mosquito	All	Infectious	Mosquito	All	Infectious	Mosquito	All	Infectious	Mosquito
*n*	20	20	160	61	16	37	106	23	282	120	31	294
Median MOI_H/M_ (IQR)	1 (1–2)	1 (1–2)	1 (1–2)	2 (1–3)	1.5 (1–3)	1 (1–2)	5 (2–8)	7 (4–11)	2 (1–3)	4 (2–7)	5 (3–8)	2 (1–3)
Allelic richness, Hs (*n* alleles)	16 (16)	16 (16)	23 (29)	26 (44)	17 (17)	22 (22)	32 (67)	29 (41)	31 (44)	34 (88)	32 (57)	36 (65)

#### 3.1.2 Allelic diversity in humans

The total number of alleles circulating in the population normalised against sample size, the allelic richness, was calculated (Table 1). The allelic richness followed the MOI_H_ and as such was highest in the human samples from Burkina Faso and lowest in the Gambian samples. Children under 15 years of age from Burkina Faso had the highest diversity of alleles in the population (Supplementary Table S2).

### 3.2 Molecular comparison of human and mosquito transmissions

#### 3.2.1 Multiplicity of infection in mosquitoes (MOI_M_) and alleles sampled

Ninety individuals successfully infected mosquitoes. The proportion of infectious individuals with monoclonal infections was 30% (27/90) and of these the majority (20/27) were from The Gambia. The number of clones in infectious versus non-infectious individuals was similar in all studies (Table 1), except in the Burkina Faso dry season, where a higher number of clones were seen in infectious individuals (*P* = 0.02). This could be related to a higher parasite density in infectious versus non-infectious MSP2-positive individuals (Supplementary Table S4).

We analysed 773 mosquitoes infected by the 90 individuals and calculated MOI_M_, the median number of clones per infected mosquito (Table 1). As with human infections, MOI_M_ was higher in Burkina Faso than in The Gambia. The median number of clones was consistently lower in individual mosquitoes than in humans. However, and somewhat surprisingly, allelic richness was higher in mosquitoes compared with the human blood they had fed on. This was observed in all studies (Table 1).

#### 3.2.2 Matching alleles in humans and mosquitoes

**Fig. 1:**
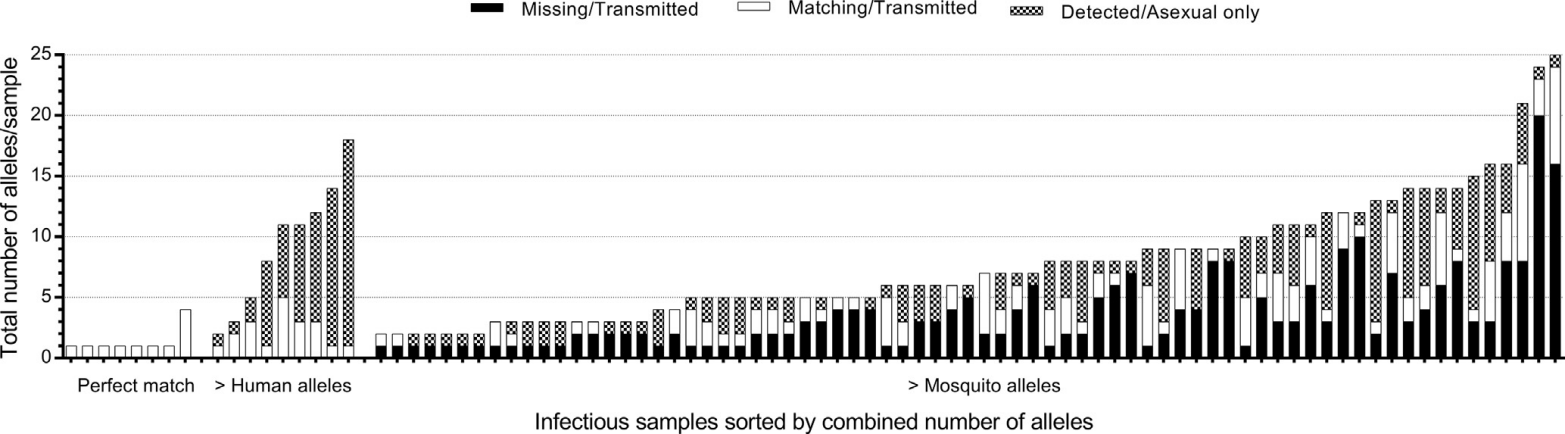
Total number of alleles in 90 infectious individuals by *Plasmodium falciparum* merozoite surface protein 2 genotyping. On the *X*-axis, the infectious individuals were grouped by matching success and sorted by number of total alleles per sample. The total number of alleles is made up of non-transmitted human alleles (Detected/Asexual only); transmitted alleles detected in the human blood samples (Matching/Transmitted) and transmitted alleles unique to mosquitoes (Missing/Transmitted).

We considered three different outcomes from the MSP2 genotyping results: perfectly matching clones (that is, all clones detected in a study participant match all clones detected in at least one mosquito infected by the same individual), more clones detected in humans than in corresponding infected mosquitoes and more clones detected in mosquitoes. In 8.9% of individuals (8/90), all MSP2 alleles detected in the humans were transmitted to the mosquitoes. In a similarly small subset of individuals (10%; 9/90) not all MSP2 alleles identified in the human blood were detected in mosquitoes. In the majority of experiments, however, we identified additional MSP2 alleles in the mosquito midguts that were not detected in the human samples on which they had fed (81.1%, 73/90). The alleles unique to mosquitoes in the individual experiments ranged between 10% and 88.9% of the total alleles in the infection (Fig. 1). Overall, the percentages of clones transmitted to mosquitoes that were not detected in the human blood were 80, 68.5, 41 and 62% in The Gambia 1994, 1999 and Burkina Faso dry and wet seasons, respectively.

One explanation of alleles being missed by MSP2 typing is preferential amplification of smaller alleles i.e. sizing bias (Supplementary Data S1). The total number of distinct allelic types detected in all blood parasites was 105 alleles. Allele frequencies varied and most alleles were present in all studies. Fifty-nine percent of the alleles belonged to the 3D7-specific allelic family and 41% belonged to the Fc27-specific allelic family. The smallest allele was 200 bp long and the largest allele was 510 bp long. Overall, 41% of all alleles sampled were smaller than 300 bp, 43% were sized between 300 and 400 bp and 16% were >400 bp. The allelic size distribution was similar in all sample types and allelic size did not affect the likelihood of detection (Supplementary Fig. S1). In mosquitoes, we detected 79 distinct alleles compared with 76 alleles in the blood of the samples the mosquitoes had fed on. In 10 out of 773 mosquitoes analysed, five allelic types not seen in any of the human blood samples were detected in the oocysts (Supplementary Table S5).

**Fig. 2:**
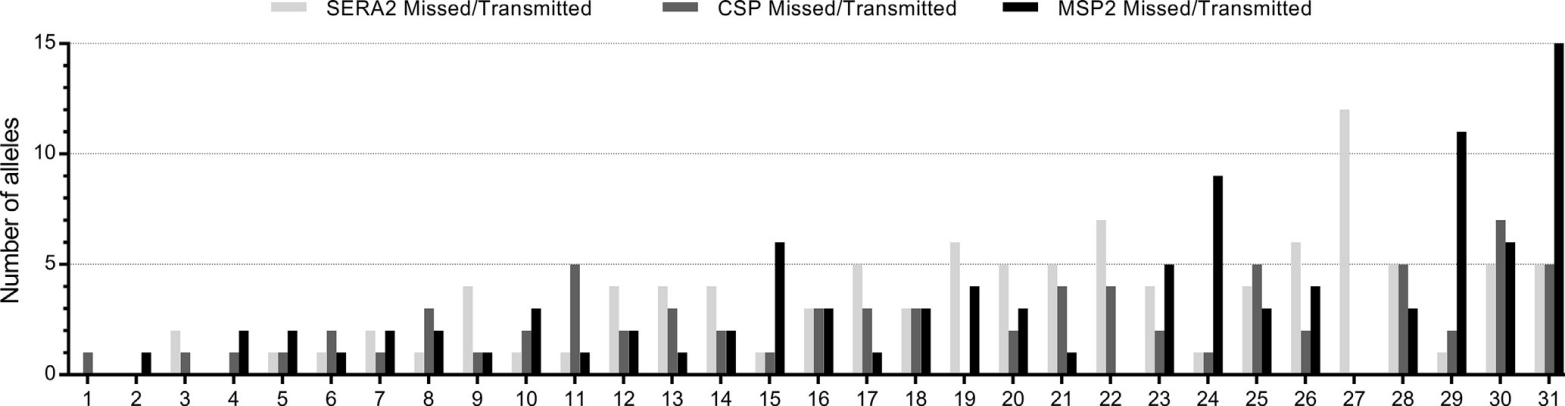
Comparison of merozoite surface protein 2 capillary electrophoresis genotyping results and circumsporozoite protein/serine repeat antigen 2 amplicon sequencing in *Plasmodium falciparum*. Number of alleles missed in human blood samples but transmitted to mosquitoes by infectious sample and marker is shown. The 31 infectious samples are numbered on the *X*-axis (1–31).

Another explanation may lie in the genotyping approach. In order to rule out that any of the unmatched alleles in the mosquitoes were assay- or target-specific, we performed next generation sequencing of PCR amplicons targeting two highly polymorphic parasite antigens, SERA2 and CSP, in a subset of mosquito and human blood samples with sufficient volume for testing. In total, 31 infectious human samples and 208 mosquito samples from the Burkina Faso surveys were tested. As with MSP2 CE genotyping data, we attempted to match mosquito parasite amplicons with those in the human blood from which they fed. Results for all three markers (MSP2, SERA2 and CSP) indicated consistency in observations of missing alleles in the human blood that are transmitted to mosquitoes (Fig. 2).

Lastly, it is possible that our genotyping in human samples was affected by asexual parasites that are typically present at considerably higher densities than sexual stage gametocytes and may affect the ability to detect all gametocyte-producing clones. We thus performed magnetic enrichment for gametocytes on a subset of whole blood samples from the Burkina Faso cross sectional surveys (106/142) to specifically genotype parasite isolates that are potentially transmissible. Genotyping was successful in 70% (75/106) of the gametocyte-enriched fractions and 80.2% (85/106) of the gametocyte-depleted samples. The median number of clones in the gametocyte fraction was 2 (interquartile range (IQR): 1, 5) and the median number of clones in the gametocyte-depleted fraction was 6 (IQR: 3, 9).

**Fig. 3:**
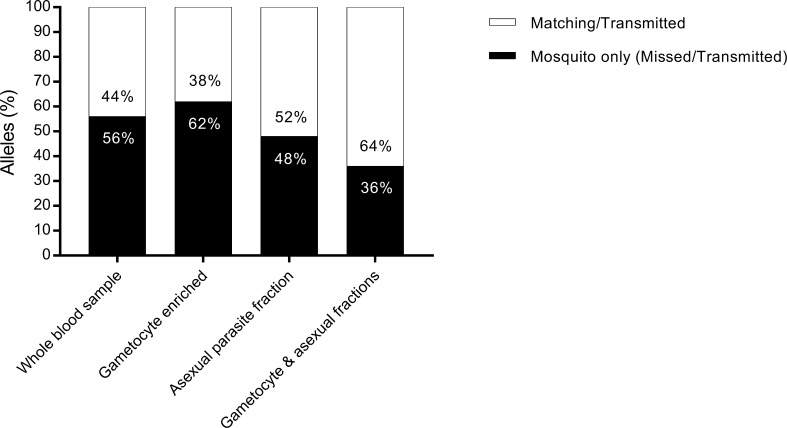
*Plasmodium falciparum* merozoite surface protein 2 genotyping results in mosquitoes and different parasite samples. Percentage of total mosquito alleles that were detected (Matching/Transmitted) and missed (Mosquito only, Missed/Transmitted) by compartment; whole blood sample, gametocyte enriched fraction, asexual parasite fraction and combined gametocyte and asexual fractions.

Magnetic enrichment data was available for 12 infectious individuals and corresponding 66 infected mosquitoes. All infectious samples had the gametocyte enriched fraction successfully genotyped. However, the 66 infected mosquitoes still contained allelic types that were not identified in the gametocyte fractions by either MSP2 CE or amplicon sequencing. Some clones unique to mosquitoes compared with whole blood were identified in the gametocyte-depleted samples. This suggests that magnetic enrichment does not fully capture all gametocyte clones (Fig. 3).

## 4 Discussion

In this observational study we investigated transmission of naturally occurring malaria clones from humans to mosquitoes. The number of clones was higher in blood and mosquito samples from Burkina Faso compared with those collected in The Gambia. In both settings, additional MSP2 alleles were consistently identified in mosquitoes compared with the human blood sample they had fed on; a finding confirmed by amplicon sequencing of CSP and SERA2. This may be due to preferential amplification of dominant (higher density) clones (Supplementary Fig. S2) but also suggests that infectious gametocytes are circulating in the human host at very low densities (Supplementary Table S6).

In the current study, MOI and allelic diversity in humans were higher in Burkina Faso during both peak and low season surveys than in The Gambia. It is likely that our findings reflect differences in transmission intensity, although sampling strategies differed between studies and make a direct comparison of MOI between sites inappropriate (Arnot, 1998). In Burkina Faso in 2013–2014, the estimated national mean parasite rate in 1–10-year olds (PfPR2-10) was approximately 40% and in The Gambia in 2000, PfPR2-10 was approximately 20% (Bhatt et al., 2015). Higher MOI and allelic richness in areas of intense malaria transmission have been reported before (Felger et al., 2012, Barry et al., 2013).

In the samples from Burkina Faso, infected children aged 5–15 years carried the highest number of clones and contributed the highest diversity of parasites to the population. This is important, as this age group constitutes a major contributor to malaria transmission (Stone et al., 2015). Interestingly, in our studies, participant age did not influence the number and the diversity of alleles transmitted to and detected in mosquitoes. This suggests that semi-immune children harbour high diversity and high density parasitaemias but not all of the parasites within the infection are transmissible at the time of sampling.

Early studies in *Plasmodium chabaudi* demonstrated that multiclonal infections are more infectious to mosquitoes (Taylor and Read, 1997). Two studies in *P. falciparum* (Nwakanma et al., 2008, Morlais et al., 2015) found that monoclonal infections lead to higher mosquito infection prevalence and intensity. Gametocyte complexity may on the other hand fuel the diversity of parasites seen in endemic populations and infection longevity, and gametocyte prevalence was previously reported to be increased in multiclonal infections (Nassir et al., 2005). Future longitudinal studies that include repeated mosquito feeding assays in monoclonal and multiclonal infections may provide further evidence on these patterns that may be of great relevance in understanding the spread of parasite populations.

At a population level, the majority of allelic types that were detected in mosquitoes were also detected in at least one of the human blood donors, however, there was limited agreement between the alleles detected in human peripheral blood and mosquitoes in individual experiments. The majority of mosquito feeds (>80%) resulted in oocysts where at least one allele was found to be unique in mosquitoes (median number of unique alleles = 2, IQR: 1–4). This may be related to the number of mosquitoes analysed, PCR sizing bias or difficulties in detecting minority genotypes in human blood where the MOI was high. We explored these different explanations. Firstly, we observed that the number of matching alleles increased with the number of mosquitoes analysed (Supplementary Fig. S3) and thus the quantity of mosquito blood meals that contributed to the comparison; we tested multiple mosquito blood meals (in the range of 2–3 µl per mosquito) versus a single 5 µl human blood sample. Multiple blood samples from the same individual, ideally collected at different time-points, would potentially result in a larger proportion of all circulating alleles being detected.

Secondly, there is also a potential for sizing bias where smaller fragments within an allelic family are more efficiently amplified than longer fragments of the same allelic family (Messerli et al., 2017). We did not observe a strong association between allele size and the likelihood that alleles were missed in human samples. In addition, amplicon sequencing results confirmed the presence of alleles below the current limit of detection of molecular methods in complex infections and this approach is unaffected by allele size.

Thirdly, preferential amplification of high density template in the presence of low density template is an acknowledged limitation of some PCRs (Walsh et al., 1992, Nwakanma et al., 2008, Koepfli et al., 2011, Wampfler et al., 2014, Morlais et al., 2015). This is particularly challenging for transmission studies where transmissible gametocytes typically comprise less than 5% of the total parasite biomass (Taylor and Read, 1997, Greischar et al., 2016). We tested magnetic enrichment of gametocytes as a more sensitive detection approach for transmissible parasites. Whilst additional alleles that resulted in mosquito infections were detected in the gametocyte-enriched fraction only, overall agreement between alleles detected in the human peripheral blood and mosquitoes remained low. One alternative approach to magnetic enrichment would have been the use of polymorphic RNA targets. Wampfler et al. (2014) used CE genotyping on gametocyte marker RNA and asexual marker DNA to describe *Plasmodium* transmission dynamics in Papua New Guinea and reported the absence of matching alleles in 14.3% and 40.5% of cases in between *pfs230* DNA/RNA and *pfg377* DNA/RNA, respectively. Our findings support this observation that many gametocyte-producing clones are undetectable by DNA-based assays and add the key observation that these undetectable clones frequently result in mosquito infections. These mosquito samples were collected on day 7 after membrane feeding, when genome copies have multiplied in individual oocysts (Bell and Ranford-Cartwright, 2004, Stone et al., 2013) and result in a more sensitive detection of all transmissible clones. In the current study where the transmission of *P. falciparum* clones from humans to mosquitoes using a combination of CE genotyping and amplicon sequencing on enriched gametocyte samples, asexual parasites and whole blood samples was investigated, we show that mosquitoes sample complex infections at a sensitivity that is unmatched by molecular diagnostics on human blood samples. The failure to detect all transmissible clones is most likely due to differences in the density of the individual clones infecting the individual and allows two important conclusions: (i) assessments of the complexity of *P. falciparum* infections that rely on samples collected at a single time-point will miss clones that are transmissible at that moment; (ii) low-density gametocyte–producing clones are capable of successfully establishing infections in mosquitoes. Future studies on transmission dynamics would benefit from approaches where parasites are genotyped in both the human and mosquito reservoir to more comprehensively characterise the circulating parasite population.

## Appendix A Supplementary data

Supplementary data 2

Supplementary data 3 Supplementary Fig. S1. *Plasmodium falciparum* alleles unique in mosquitoes and matched in mosquitoes by allele size. (A) Proportion of unique mosquito alleles and alleles matched in mosquitoes and the human blood they had fed on, by allele size and allelic family (3D7 allelic family). (B) Proportion of unique mosquito alleles and alleles matched in mosquitoes and the human blood they had fed on, by allele size and allelic family (Fc27 allelic family). Supplementary Fig. S2. Number of *Plasmodium falciparum* clones transmitted to mosquitoes that remained undetected in the human blood on which the mosquitoes fed, by asexual parasite density (A) and gametocyte density (B). Supplementary Fig. S3. Unique *Plasmodium falciparum* alleles in mosquitoes per feeding assay by number of mosquitoes analysed.
