# Supplementary material for: Transmission of molecularly undetectable circulating parasite clones leads to high infection complexity in mosquitoes post feeding
Source: Int J Parasitol. 2018 Jul;48(8):671–7. doi: 10.1016/j.ijpara.2018.02.005 (PMC6018601; doi:10.1016/j.ijpara.2018.02.005)
Supplement: Supplementary data 1 [file mmc1.docx]

**Supplementary Data S1**

**1. Supplementary materials and methods**

Control samples were prepared from *Plasmodium falciparum* laboratory strains in order to test the limit of detection (LOD) of the merozoite surface protein 2 (MSP2) genotyping assay. A dilution series (2000, 1000, 500, 50, 5, 1 and 0.1 parasites/µl) of cultured NF54 and NF135 strains was used to test the LOD in whole blood samples. In addition, NF54 and NF135 strains were fed to mosquitoes by standard membrane feeding assay (SMFA) (([Bousema et al., 2012](#_ENREF_8)) using the following NF54/NF135 ratios: 100%:0%; 75%:25%; 50%:50%; 25%:75% and 0%:100%. Oocyst-positive samples were collected on day 7 and extracted using the phenol/chloroform method. To test whether we can detect different concentrations of clones in one sample, DNA from the laboratory strains (5 ng of NF54, NF135, NF166, NF165 and NF175) was diluted 1 in 100 and mixed at ratios containing 80% or 60% of one of the five strains and 5% or 10% of the other four strains, respectively. All ratio samples were prepared in duplicate and each reaction done in triplicate.

**2. Supplementary results - LOD**

We tested the LOD of the assay on cultured parasites (NF54 and NF135) and mosquito oocysts. We were able to detect 0.1 parasites/µl of cultured parasites in whole blood. In mosquito oocyst samples infected with NF54 and NF135, both strains were detected at different input ratios. However, the NF135 allele was missed in one of the four low ratio samples (75% NF54:25% NF135). Next, we tested samples containing different ratios of five cultured strains, NF54, NF135, NF166, NF165 and NF175. All parasite clones were consistently identified, independent of ratio of input DNA. NF175 belongs to the Fc27 allelic family and the other four strains belong to the 3D7 allelic family. The largest 3D7 specific MSP2 allele, NF166/363 (363 bp fragment), did not amplify as efficiently and the real fluorescence units (RFU) fell below the arbitrary threshold of 500 RFU. This was not observed with the 411 bp Fc27-specific fragment in NF175 samples.
