# Supplementary material for: Transmission of molecularly undetectable circulating parasite clones leads to high infection complexity in mosquitoes post feeding
Source: Int J Parasitol. 2018 Jul;48(8):671–7. doi: 10.1016/j.ijpara.2018.02.005 (PMC6018601; doi:10.1016/j.ijpara.2018.02.005)
Supplement: Supplementary data 2 [file mmc2.docx]

**Supplementary Table S1.** Study characteristics

| **Study** | **Gambia 1994** | **Gambia 1999** | **Burkina Faso (dry)** | **Burkina Faso (wet)** |
| --- | --- | --- | --- | --- |
| Participants | Children and adults | Children | Children and adults | Children and adults |
| Enrolment criteria - parasites | Yes^a^ | Yes^b^ | Not tested^c^ | Not tested^c^ |
| Drug treatment | No | Yes^d^ | No | No |
| Status | Infectious only | Infectious & non-infectious | Infectious & non-infectious | Infectious & non-infectious |
| MSP2 CE positives (*n*) | 20 | 61 | 106 | 120 |
| Parasite density  (by microscopy) | 1990.6 (95% CI 355.3-11152.4;  *n* =12) | 67.1 (*n*=2) | 799.1 (95% CI 546.9-1167.8;  *n* =47) | 1197.9 (95% CI 822.6-1744.6; *n*=93) |
| Gametocyte density  (by microscopy) | 171.8 (95% CI 82.2-359.0; *n*=20) | 8.1 (95% CI 4.8-13.6; *n*=47) | 39.1 (95% CI 17.9-85.5; *n*=11) | 40.5 (95% CI 29.1-56.4; *n*=57) |
| Assays | MSP2 CE | MSP2 CE | MSP2 CE  Amplicon sequencing | MSP2 CE  Magnetic enrichment^e^  Amplicon sequencing |

^a^ Individuals presenting at health facility carrying *Plasmodium falciparum* gametocytes were specifically chosen

^b^ Individuals presenting at health facility

^c^ Cross-sectional surveys – asymptomatic individuals enrolled

^d^ Two treatment arms: Pyrimethamine sulfadoxine (PSD) or PSD + three doses of artesunate (AS). Treatment on Day 0 and samples collection and membrane feeds on Day7

^e^ Subset of 13 infectious blood samples and positive mosquitoes have magnetic enrichment data for merozoite surface protein 2 (MSP2) capillary electrophoresis (CE) and amplicon sequencing.

CI, Confidence interval.

**Supplementary Table S2.** Multiplicity of infection (MOI_H_), allelic richness (Hs) and number of alleles sampled by age of all *Plasmodium falciparum* merozoite surface protein 2 (MSP2) positive individuals from Burkina Faso during the dry and wet seasons. [Table must ‘stand alone’ so the name of the parasite must be mentioned somewhere in the legend, table or notes.]

|  | **Burkina Faso (dry season)** | |  | **Burkina Faso (wet season)** | |
| --- | --- | --- | --- | --- | --- |
| **Age (years)** | **MOI_H_** | **Allelic richness, Hs (*n*. of alleles)** | | **MOI_H_** | **Allelic richness, Hs *(n*. of alleles)** |
| <5 | 4 (2-8), *n*=21 | 38.2 (45) |  | 4 (2-6), *n*=19 | 38.5 (44) |
| 5-15 | 5 (3.5-9), *n*=68 | 40.0 (61) |  | 5 (3-9), *n*=73 | 46.6 (87) |
| >15 | 2 (1-4), *n*=17 | 29 (29) |  | 2 (1.5-4), *n*=28 | 34.2 (38) |

**Supplementary Table S3. *Plasmodium falciparum*** multiplicity of infection (MOI_H/M_) in The Gambia 1999 study by treatment arm, Pyrimethamine sulfadoxine (PSD) or PSD + three doses of artesunate (AS).

| **The Gambia 1999** | **PSD** | **PSD + three x AS** |
| --- | --- | --- |
| MOI_H_ (IQR), all humans | 1 (1-3) | 2 (1-3) |
| MOI_H_ (IQR), infectious only | 1 (1-2) | 2 (1-3) |
| MOI_M_ (IQR), mosquitoes | 2 (1-3) | 2 (1-3) |
| Unique mosquito alleles (%) | 71% | 66% |

IQR, interquartile range.

**Supplementary Table S4.** Asexual *Plasmodium falciparum* parasite density by season and infectivity in Burkina Faso. Geometric mean in merozoite surface protein 2 (MSP2) positive individuals that were either infectious or non-infectious to mosquitoes.

|  | **Burkina Faso (dry season)** | |  | **Burkina Faso (wet season)** | |
| --- | --- | --- | --- | --- | --- |
|  | **Non-infectious** | **Infectious** | | **Non-infectious** | **Infectious** |
| Number of MSP2 positive samples | *n*=33 | *n*=14 |  | *n*=64 | *n*=29 |
| Asexual parasite density (by microscopy) | 616.4 (95% CI, 375.8-1011.0) | 1473.9 (95% CI, 964.2-2252.8) |  | 890.0 (95% CI, 579.8-1366.0) | 2308.3 (95% CI, 1113.0 – 4787.4) |

CI, Confidence interval.

**Supplementary Table S5.** *Plasmodium falciparum* allele frequency in infected humans (INFECT H), in all merozoite surface protein 2 (MSP2) positive human (H) samples (ALL) and mosquitoes (M) by study. Alleles highlighted in red where exclusively found in mosquito midguts.

| **Allele** | **G94 INFECT (H)** | **G94 (M)** | **G99 INFECT (H)** | **G99 ALL** | **G99 (M)** | **BF (WET) INFECT (H)** | **BF (WET) ALL** | **BF (WET, M)** | **BF (DRY) INFECT H** | **BF (DRY) ALL** | **BF (DRY, M)** |
| --- | --- | --- | --- | --- | --- | --- | --- | --- | --- | --- | --- |
| 3D7_200 |  |  |  | 0.73 |  | 0.57 | 0.32 |  |  |  |  |
| 3D7_205 |  | 3.16 |  | 0.73 |  | 0.57 | 0.32 | 1.91 | 1.12 | 0.35 | 1.04 |
| Fc27_209 |  |  |  |  |  | 0.57 | 0.48 |  |  |  |  |
| 3D7_210 |  |  |  |  |  | 0.57 | 0.65 | 0.48 |  | 0.53 |  |
| 3D7_214 |  |  |  |  |  |  | 0.32 | 0.48 |  |  |  |
| 3D7_218 |  |  |  |  |  | 1.72 | 1.13 | 0.96 | 1.12 | 0.7 | 2.08 |
| Fc27_219 |  | 1.05 |  |  |  | 1.72 | 2.26 | 2.39 | 1.12 | 2.81 | 1.04 |
| 3D7_223 |  | 9.47 |  | 3.65 | 2.94 | 1.15 | 0.97 | 1.44 | 2.25 | 0.53 | 1.04 |
| 3D7_226 |  |  |  |  |  |  |  |  |  | 0.53 |  |
| 3D7_230 |  |  |  |  |  | 2.3 | 1.29 | 1.91 | 1.12 | 2.81 | 2.08 |
| 3D7_233 | 3.23 |  | 6.45 | 1.46 |  | 1.15 | 0.32 | 0.48 |  |  |  |
| 3D7_233 | 3.23 | 3.16 | 9.68 | 3.65 | 2.94 | 1.72 | 1.77 | 1.44 | 1.12 | 1.05 | 3.13 |
| Fc27_238 |  |  |  |  |  |  |  | 0.48 |  |  |  |
| 3D7_240 |  |  |  | 0.73 |  |  | 1.13 | 0.48 | 1.12 | 1.93 | 1.04 |
| 3D7_243 |  | 1.05 |  | 0.73 |  | 1.15 | 0.81 | 1.44 | 2.25 | 0.53 |  |
| 3D7_246 |  |  |  |  |  | 0.57 | 0.16 |  |  | 1.76 |  |
| 3D7_249 |  |  |  |  |  | 0.57 | 1.29 | 1.91 | 1.12 | 1.58 | 3.13 |
| 3D7_253 |  |  |  | 1.46 |  | 1.72 | 1.13 | 2.87 | 2.25 | 1.41 | 2.08 |
| 3D7_256 |  |  |  |  |  |  | 0.97 | 0.48 |  |  |  |
| Fc27_257 |  | 2.11 |  | 0.73 |  | 1.72 | 0.97 | 2.39 | 1.12 | 2.28 | 3.13 |
| 3D7_260 |  | 2.11 |  | 0.73 |  | 4.6 | 3.39 | 3.83 | 8.99 | 2.81 | 5.21 |
| 3D7_263 |  |  |  |  |  | 0.57 | 1.13 | 0.48 |  |  |  |
| 3D7_266 | 9.68 | 11.58 | 6.45 | 5.84 |  | 7.47 | 5.48 | 5.26 |  | 3.16 | 3.13 |
| 3D7_270 | 3.23 | 2.11 |  | 1.46 |  | 4.6 | 3.39 | 4.31 | 2.25 | 4.22 | 4.17 |
| 3D7_275 |  | 1.05 |  | 0.73 | 2.94 | 1.15 | 2.26 | 2.87 | 2.25 | 2.11 | 2.08 |
| 3D7_278 |  |  |  |  |  | 2.3 | 1.29 | 1.44 |  |  |  |
| 3D7_281 |  |  | 3.23 | 2.92 | 2.94 | 2.87 | 1.94 | 1.44 | 1.12 | 2.11 | 2.08 |
| Fc27_283 |  |  |  |  | 2.94 | 0.57 | 0.16 |  |  | 0.35 |  |
| 3D7_284 | 3.23 |  |  | 1.46 |  | 2.3 | 1.61 | 2.87 |  | 0.88 |  |
| 3D7_287 |  |  |  |  |  | 2.87 | 1.29 | 2.87 | 1.12 | 0.88 |  |
| 3D7_290 |  | 1.05 |  |  |  |  | 0.32 |  |  | 3.51 |  |
| Fc27_291 | 3.23 | 4.21 |  | 2.92 | 2.94 | 1.72 | 1.61 | 1.91 | 1.12 | 1.76 | 4.17 |
| 3D7_293 |  |  |  |  |  | 2.3 | 4.03 | 2.39 |  |  |  |
| 3D7_294 |  | 1.05 |  | 0.73 |  |  |  |  | 5.62 | 1.76 | 5.21 |
| 3D7_296 | 3.23 | 1.05 |  | 0.73 |  | 0.57 | 0.97 | 1.91 |  |  |  |
| Fc27_297 |  |  |  |  |  |  | 0.16 |  |  |  |  |
| 3D7_299 | 3.23 | 3.16 | 9.68 | 5.11 | 5.88 |  | 1.29 |  | 1.12 | 0.88 | 1.04 |
| Fc27_301 |  |  |  |  |  | 0.57 | 0.48 |  |  | 0.35 |  |
| 3D7_303 |  | 3.16 |  |  |  |  |  | 0.48 |  | 0.7 |  |
| 3D7_306 |  | 1.05 |  |  |  | 0.57 | 0.65 | 0.48 |  | 1.76 | 1.04 |
| Fc27_307 |  |  |  |  |  | 0.57 | 0.48 |  |  | 0.18 |  |
| 3D7_310 |  |  |  | 0.73 |  | 3.45 | 1.77 | 1.91 | 1.12 | 1.93 | 1.04 |
| Fc27_311 |  |  |  |  |  |  | 0.16 | 0.96 | 1.12 | 0.35 |  |
| 3D7_313 |  |  |  |  |  | 1.15 | 1.29 | 2.39 |  |  |  |
| Fc27_315 |  | 2.11 |  | 0.73 |  | 2.87 | 2.26 | 1.91 | 1.12 | 2.46 |  |
| 3D7_316 |  |  |  | 1.46 | 2.94 |  | 0.48 | 0.48 | 3.37 | 1.93 | 4.17 |
| 3D7_319 |  |  |  |  |  |  | 1.29 |  | 1.12 | 1.58 | 1.04 |
| Fc27_320 |  |  |  |  |  |  | 0.48 | 0.96 |  |  |  |
| Fc27_323 |  |  |  |  |  |  |  | 0.96 |  |  |  |
| 3D7_324 |  | 6.32 | 3.23 | 0.73 | 5.88 | 0.57 | 0.32 | 2.39 | 4.49 | 1.23 | 4.17 |
| 3D7_327 |  |  |  |  |  | 1.15 | 0.65 | 2.39 |  |  |  |
| Fc27_328 | 16.13 | 8.42 | 12.9 | 16.06 | 5.88 | 10.92 | 10.65 | 3.83 | 13.48 | 9.31 | 11.46 |
| 3D7_330 |  |  |  | 0.73 | 2.94 |  | 0.65 | 0.48 | 1.12 | 1.05 | 2.08 |
| 3D7_333 |  |  | 6.45 | 2.19 | 2.94 | 0.57 | 1.13 |  |  | 1.23 |  |
| Fc27_334 |  | 3.16 |  | 0.73 |  |  |  |  |  |  |  |
| 3D7_336 |  |  |  |  |  |  | 0.32 |  |  |  |  |
| Fc27_339 |  |  |  |  |  |  | 0.16 |  |  |  |  |
| 3D7_340 |  |  | 3.23 | 1.46 | 8.82 |  | 0.48 | 0.96 |  | 0.53 |  |
| Fc27_342 |  |  |  |  |  | 1.15 | 0.48 |  |  | 0.18 | 1.04 |
| 3D7_343 |  |  |  |  |  |  | 0.32 | 0.96 |  | 0.18 |  |
| 3D7_346 |  | 3.16 |  | 0.73 | 5.88 | 1.15 | 0.81 | 1.44 | 1.12 | 1.58 | 1.04 |
| Fc27_347 |  |  |  |  |  |  | 0.16 | 0.96 |  |  |  |
| 3D7_350 |  | 1.05 |  | 0.73 |  | 1.15 | 0.65 | 0.48 |  |  | 1.04 |
| Fc27_352 |  |  |  |  |  | 1.15 | 1.77 | 1.91 | 1.12 | 1.58 | 1.04 |
| 3D7_353 | 3.23 |  |  | 0.73 | 5.88 |  |  |  |  | 0.53 |  |
| Fc27_354 |  |  | 3.23 | 1.46 |  |  |  |  |  |  |  |
| 3D7_357 | 3.23 | 6.32 | 3.23 | 1.46 | 2.94 | 1.72 | 2.1 | 2.87 | 2.25 | 1.58 | 1.04 |
| Fc27_359 |  |  |  |  |  |  | 0.16 |  |  |  |  |
| 3D7_363 |  |  |  |  |  | 0.57 | 0.48 |  |  | 1.05 |  |
| Fc27_365 | 9.68 | 7.37 | 6.45 | 7.3 | 11.76 | 4.02 | 5.32 | 2.87 | 3.37 | 6.5 | 5.21 |
| 3D7_368 |  |  | 3.23 | 0.73 | 2.94 | 0.57 | 0.32 | 0.48 |  | 0.88 | 1.04 |
| Fc27_370 |  |  |  |  |  |  | 0.48 |  | 2.25 | 0.53 | 1.04 |
| 3D7_373 |  |  |  |  |  |  | 1.13 | 0.48 | 2.25 | 1.41 | 1.04 |
| Fc27_376 |  |  |  |  |  | 0.57 | 0.97 |  |  | 0.18 |  |
| 3D7_377 |  |  |  |  |  |  |  |  | 1.12 | 0.18 |  |
| 3D7_380 |  |  |  |  |  | 0.57 | 0.32 | 0.96 |  | 1.05 |  |
| Fc27_382 |  |  |  |  |  |  |  |  |  | 0.35 |  |
| 3D7_383 |  | 1.05 | 3.23 | 0.73 | 2.94 |  | 0.16 |  |  |  |  |
| 3D7_386 |  |  |  |  |  |  | 0.32 |  | 1.12 | 0.18 | 1.04 |
| Fc27_387 |  |  |  | 0.73 |  |  |  |  | 1.12 | 0.18 |  |
| 3D7_390 |  |  |  | 0.73 |  |  |  |  |  |  |  |
| 3D7_391 |  |  |  |  |  | 1.15 | 1.13 | 3.35 |  | 1.05 | 1.04 |
| Fc27_392 |  |  |  |  |  |  | 0.32 | 0.48 |  |  |  |
| 3D7_397 |  |  |  |  |  | 0.57 | 0.32 | 0.48 |  |  | 1.04 |
| Fc27_400 | 6.45 | 2.11 |  |  |  |  | 0.48 | 0.48 | 4.49 | 1.58 | 3.13 |
| 3D7_402 |  |  |  |  |  |  | 0.48 |  |  | 0.18 |  |
| 3D7_408 |  |  |  |  |  | 0.57 | 0.32 | 0.48 | 1.12 | 0.18 |  |
| Fc27_411 | 22.58 | 6.32 | 12.9 | 16.79 | 8.82 | 6.9 | 7.58 | 3.35 | 10.11 | 9.49 | 3.13 |
| 3D7_415 |  |  |  | 1.46 |  |  |  | 0.48 |  |  |  |
| 3D7_422 |  |  |  |  |  |  |  |  |  |  | 1.04 |
| 3D7_427 |  |  |  |  |  |  | 0.32 |  |  | 0.18 |  |
| Fc27_428 |  |  |  |  |  |  | 0.32 |  |  |  |  |
| 3D7_430 | 3.23 |  | 3.23 | 0.73 |  | 0.57 | 0.16 | 0.48 |  |  | 1.04 |
| 3D7_433 |  |  | 3.23 | 2.19 | 2.94 |  | 0.16 | 0.48 |  | 0.18 |  |
| Fc27_434 |  |  |  |  |  | 1.15 | 0.32 |  |  |  |  |
| Fc27_441 |  |  |  | 0.73 |  |  | 0.48 |  |  |  |  |
| Fc27_448 | 3.23 | 1.05 |  |  |  | 1.15 | 0.48 | 0.96 | 1.12 | 0.7 |  |
| 3D7_456 |  |  |  |  |  |  | 0.16 | 0.48 | 1.12 | 0.35 | 1.04 |
| 3D7_459 |  |  |  |  |  | 0.57 | 0.16 |  |  |  |  |
| 3D7_463 |  |  |  |  |  | 0.57 | 0.16 |  |  |  |  |
| Fc27_464 |  |  |  |  |  |  | 0.16 |  |  |  |  |
| 3D7_467 |  |  |  |  |  |  |  | 0.96 |  |  |  |
| Fc27_469 |  |  |  |  |  |  |  |  |  | 0.18 |  |
| 3D7_499 |  |  |  |  |  |  |  |  |  |  | 1.04 |
| Fc27_510 |  |  |  | 1.46 | 2.94 |  | 0.16 | 0.48 |  |  | 1.04 |

G94, The Gambia 1994; G99, The Gambia 1999; BF, Burkina Faso; WET, wet season; DRY, dry season.

**Supplementary Table S6.** *Plasmodium falciparum* merozoite surface protein 2 (MSP2) positive samples, multiplicity of infection (MOI), asexual parasite and gametocyte densities (p/µl). Infection prevalence is the proportion infected mosquitoes among dissected mosquitoes. Both infectious and non-infectious samples are sorted by MOI.

| **ID** | **MOI** | **Asexual parasites (p/µl)** | **Gametocytes (p/µl)** | **Log10 asexual parasites (p/µl)** | **Log 10 gametocytes (p/µl)** |
| --- | --- | --- | --- | --- | --- |
| Gambia94_1 | 1 |  | 50 | 0 | 1.70757 |
| Gambia94_2 | 1 |  | 500 | 0 | 2.69984 |
| Gambia94_4 | 1 |  | 25 | 0 | 1.41497 |
| Gambia94_7 | 1 |  | 120 | 0 | 2.08279 |
| Gambia94_8 | 1 | 4500 | 410 | 3.65331 | 2.61384 |
| Gambia94_10 | 1 |  | 1000 | 0 | 3.00043 |
| Gambia94_12 | 1 | 14,000 | 500 | 4.14616 | 2.69984 |
| Gambia94_15 | 1 |  | 10 | 0 | 1.04139 |
| Gambia94_16 | 1 |  | 110 | 0 | 2.04532 |
| Gambia94_17 | 1 | 20,000 | 160 | 4.30105 | 2.20683 |
| Gambia94_18 | 1 | 15 | 15 | 1.20412 | 1.20412 |
| Gambia94_20 | 1 |  | 70 | 0 | 1.85126 |
| Gambia99_1 | 1 | 26 | 0 | 1.43136 | 0 |
| Gambia99_2 | 1 | 50 | 0 | 1.70757 | 0 |
| Gambia99_8 | 1 | 100 | 0 | 2.00432 | 0 |
| Gambia99_9 | 1 | 5 | 0 | 0.778151 | 0 |
| Gambia99_11 | 1 | 2 | 0 | 0.477121 | 0 |
| Gambia99_13 | 1 | 25 | 0 | 1.41497 | 0 |
| Gambia99_14 | 1 | 150 | 0 | 2.17898 | 0 |
| Gambia99_15 | 1 | 2 | 1 | 0.477121 | 0.30103 |
| Gambia99_18 | 1 | 6 | 0 | 0.845098 | 0 |
| Gambia99_19 | 1 | 100 | 0 | 2.00432 | 0 |
| Gambia99_20 | 1 | 180 | 0 | 2.25768 | 0 |
| Gambia99_23 | 1 | 4 | 1 | 0.69897 | 0.30103 |
| Gambia99_25 | 1 | 65 | 0 | 1.81954 | 0 |
| Gambia99_26 | 1 | 75 | 0 | 1.88081 | 0 |
| Gambia99_27 | 1 | 150 | 0 | 2.17898 | 0 |
| Gambia99_28 | 1 | 100 | 0 | 2.00432 | 0 |
| Gambia99_31 | 1 | 100 | 0 | 2.00432 | 0 |
| Gambia99_36 | 1 | 250 | 0 | 2.39967 | 0 |
| Gambia99_37 | 1 | 20 | 2 | 1.32222 | 0.477121 |
| Gambia99_38 | 1 | 65 | 2 | 1.81954 | 0.477121 |
| Gambia99_39 | 1 | 75 | 0 | 1.88081 | 0 |
| Gambia99_44 | 1 | 20 | 0 | 1.32222 | 0 |
| Gambia99_45 | 1 | 50 | 0 | 1.70757 | 0 |
| Gambia99_46 | 1 | 200 | 0 | 2.3032 | 0 |
| Gambia99_51 | 1 | 40 | 0 | 1.61278 | 0 |
| Gambia99_59 | 1 | 40 | 0 | 1.61278 | 0 |
| Gambia99_60 | 1 | 450 | 22 | 2.65418 | 1.36173 |
| BurkinaDry_3 | 1 | 0 | 0 | 0 | 0 |
| BurkinaDry_4 | 1 | 0 | 0 | 0 | 0 |
| BurkinaDry_11 | 1 | 8955 | 0 | 3.95211 | 0 |
| BurkinaDry_14 | 1 | 0 | 0 | 0 | 0 |
| BurkinaDry_16 | 1 | 0 | 0 | 0 | 0 |
| BurkinaDry_32 | 1 | 0 | 0 | 0 | 0 |
| BurkinaDry_36 | 1 | 738 | 72 | 2.86864 | 1.86332 |
| BurkinaDry_39 | 1 | 0 | 0 | 0 | 0 |
| BurkinaDry_42 | 1 | 0 | 0 | 0 | 0 |
| BurkinaDry_46 | 1 | 0 | 0 | 0 | 0 |
| BurkinaDry_48 | 1 | 0 | 0 | 0 | 0 |
| BurkinaDry_54 | 1 | 0 | 0 | 0 | 0 |
| BurkinaDry_55 | 1 | 0 | 0 | 0 | 0 |
| BurkinaDry_67 | 1 | 0 | 0 | 0 | 0 |
| BurkinaDry_74 | 1 | 0 | 0 | 0 | 0 |
| BurkinaDry_77 | 1 | 0 | 0 | 0 | 0 |
| BurkinaDry_84 | 1 | 0 | 0 | 0 | 0 |
| BurkinaDry_90 | 1 | 0 | 0 | 0 | 0 |
| BurkinaDry_95 | 1 | 0 | 0 | 0 | 0 |
| BurkinaDry_102 | 1 | 0 | 0 | 0 | 0 |
| BurkinaWet_8 | 1 | 0 | 0 | 0 | 0 |
| BurkinaWet_11 | 1 | 968 | 0 | 2.98632 | 0 |
| BurkinaWet_17 | 1 | 1036 | 0 | 3.01578 | 0 |
| BurkinaWet_21 | 1 | 0 | 4 | 0 | 0.69897 |
| BurkinaWet_22 | 1 | 0 | 0 | 0 | 0 |
| BurkinaWet_34 | 1 | 786 | 0 | 2.89597 | 0 |
| BurkinaWet_36 | 1 | 0 | 0 | 0 | 0 |
| BurkinaWet_41 | 1 | 0 | 0 | 0 | 0 |
| BurkinaWet_59 | 1 | 0 | 12 | 0 | 1.11394 |
| BurkinaWet_62 | 1 | 0 | 0 | 0 | 0 |
| BurkinaWet_70 | 1 | 0 | 12 | 0 | 1.11394 |
| BurkinaWet_72 | 1 | 0 | 0 | 0 | 0 |
| BurkinaWet_78 | 1 | 0 | 0 | 0 | 0 |
| BurkinaWet_90 | 1 | 118 | 118 | 2.07555 | 2.07555 |
| BurkinaWet_98 | 1 | 0 | 64 | 0 | 1.81291 |
| BurkinaWet_102 | 1 | 40,457 | 128 | 4.607 | 2.11059 |
| BurkinaWet_112 | 1 | 13,741.5 | 661 | 4.13807 | 2.82086 |
| Gambia94_3 | 2 |  | 500 | 0 | 2.69984 |
| Gambia94_6 | 2 |  | 2000 | 0 | 3.30125 |
| Gambia94_13 | 2 |  | 125 | 0 | 2.10037 |
| Gambia94_14 | 2 | 11,500 | 100 | 4.06074 | 2.00432 |
| Gambia94_19 | 2 |  | 2500 | 0 | 3.39811 |
| Gambia99_5 | 2 | 64 | 0 | 1.81291 | 0 |
| Gambia99_12 | 2 | 150 | 0 | 2.17898 | 0 |
| Gambia99_21 | 2 | 66 | 0 | 1.82607 | 0 |
| Gambia99_22 | 2 | 15 | 0 | 1.20412 | 0 |
| Gambia99_30 | 2 | 25 | 0 | 1.41497 | 0 |
| Gambia99_33 | 2 | 150 | 0 | 2.17898 | 0 |
| Gambia99_35 | 2 | 300 | 0 | 2.47857 | 0 |
| Gambia99_41 | 2 | 200 | 0 | 2.3032 | 0 |
| Gambia99_42 | 2 | 0.2 | 0 | 0.079181 | 0 |
| Gambia99_47 | 2 | 63 | 0 | 1.80618 | 0 |
| Gambia99_48 | 2 | 75 | 3 | 1.88081 | 0.60206 |
| Gambia99_57 | 2 | 22 | 0 | 1.36173 | 0 |
| Gambia99_58 | 2 | 2 | 0 | 0.477121 | 0 |
| BurkinaDry_7 | 2 | 0 | 0 | 0 | 0 |
| BurkinaDry_15 | 2 | 0 | 64 | 0 | 1.81291 |
| BurkinaDry_23 | 2 | 269 | 0 | 2.43136 | 0 |
| BurkinaDry_35 | 2 | 3215 | 0 | 3.50732 | 0 |
| BurkinaDry_40 | 2 | 0 | 0 | 0 | 0 |
| BurkinaDry_47 | 2 | 0 | 0 | 0 | 0 |
| BurkinaDry_56 | 2 | 0 | 0 | 0 | 0 |
| BurkinaDry_86 | 2 | 0 | 0 | 0 | 0 |
| BurkinaDry_91 | 2 | 0 | 0 | 0 | 0 |
| BurkinaDry_92 | 2 | 0 | 0 | 0 | 0 |
| BurkinaDry_101 | 2 | 0 | 0 | 0 | 0 |
| BurkinaWet_1 | 2 | 0 | 16 | 0 | 1.23045 |
| BurkinaWet_5 | 2 | 370 | 0 | 2.56937 | 0 |
| BurkinaWet_10 | 2 | 0 | 0 | 0 | 0 |
| BurkinaWet_18 | 2 | 1237 | 0 | 3.09272 | 0 |
| BurkinaWet_20 | 2 | 0 | 0 | 0 | 0 |
| BurkinaWet_25 | 2 | 0 | 0 | 0 | 0 |
| BurkinaWet_27 | 2 | 71 | 0 | 1.85733 | 0 |
| BurkinaWet_33 | 2 | 0 | 0 | 0 | 0 |
| BurkinaWet_35 | 2 | 0 | 24 | 0 | 1.39794 |
| BurkinaWet_58 | 2 | 0 | 0 | 0 | 0 |
| BurkinaWet_63 | 2 | 0 | 0 | 0 | 0 |
| BurkinaWet_68 | 2 | 1170 | 0 | 3.06856 | 0 |
| BurkinaWet_81 | 2 | 0 | 8 | 0 | 0.954243 |
| BurkinaWet_93 | 2 | 517 | 39 | 2.71433 | 1.60206 |
| BurkinaWet_99 | 2 | 2218 | 16 | 3.34616 | 1.23045 |
| BurkinaWet_100 | 2 | 199 | 160 | 2.30103 | 2.20683 |
| BurkinaWet_103 | 2 | 31,921 | 64 | 4.50409 | 1.81291 |
| BurkinaWet_105 | 2 | 94 | 125 | 1.97772 | 2.10037 |
| BurkinaWet_108 | 2 | 11,528 | 63 | 4.06179 | 1.80618 |
| BurkinaWet_120 | 2 | 26,823 | 37 | 4.42852 | 1.57978 |
| Gambia94_5 | 3 |  | 25 | 0 | 1.41497 |
| Gambia94_9 | 3 |  | 420 | 0 | 2.62428 |
| Gambia94_11 | 3 | 75 | 540 | 1.88081 | 2.7332 |
| Gambia99_3 | 3 | 20 | 5 | 1.32222 | 0.778151 |
| Gambia99_4 | 3 | 45 | 0 | 1.66276 | 0 |
| Gambia99_6 | 3 | 100 | 3 | 2.00432 | 0.60206 |
| Gambia99_7 | 3 | 50 | 0 | 1.70757 | 0 |
| Gambia99_10 | 3 | 7 | 0 | 0.90309 | 0 |
| Gambia99_16 | 3 | 65 | 0 | 1.81954 | 0 |
| Gambia99_17 | 3 | 25 | 1 | 1.41497 | 0.30103 |
| Gambia99_40 | 3 | 8 | 2 | 0.954243 | 0.477121 |
| Gambia99_49 | 3 | 100 | 4 | 2.00432 | 0.69897 |
| Gambia99_50 | 3 | 35 | 0 | 1.5563 | 0 |
| Gambia99_54 | 3 | 72 | 0 | 1.86332 | 0 |
| Gambia99_61 | 3 | 260 | 0 | 2.41664 | 0 |
| BurkinaDry_1 | 3 | 0 | 0 | 0 | 0 |
| BurkinaDry_8 | 3 | 0 | 0 | 0 | 0 |
| BurkinaDry_20 | 3 | 0 | 0 | 0 | 0 |
| BurkinaDry_57 | 3 | 0 | 0 | 0 | 0 |
| BurkinaDry_65 | 3 | 0 | 0 | 0 | 0 |
| BurkinaDry_89 | 3 | 0 | 0 | 0 | 0 |
| BurkinaDry_94 | 3 | 204 | 0 | 2.31175 | 0 |
| BurkinaDry_96 | 3 | 161 | 0 | 2.20952 | 0 |
| BurkinaWet_29 | 3 | 136 | 0 | 2.13672 | 0 |
| BurkinaWet_47 | 3 | 112 | 0 | 2.05308 | 0 |
| BurkinaWet_49 | 3 | 32 | 16 | 1.51851 | 1.23045 |
| BurkinaWet_75 | 3 | 0 | 0 | 0 | 0 |
| BurkinaWet_80 | 3 | 658 | 0 | 2.81889 | 0 |
| BurkinaWet_87 | 3 | 31 | 125 | 1.50515 | 2.10037 |
| BurkinaWet_96 | 3 | 16 | 62 | 1.23045 | 1.79934 |
| BurkinaWet_104 | 3 | 1864 | 16 | 3.27068 | 1.23045 |
| BurkinaWet_111 | 3 | 17,666 | 229 | 4.24716 | 2.36173 |
| BurkinaWet_113 | 3 | 21,243.5 | 56 | 4.32725 | 1.75587 |
| BurkinaWet_118 | 3 | 835 | 112 | 2.92221 | 2.05308 |
| BurkinaWet_119 | 3 | 723 | 144 | 2.85974 | 2.16137 |
| Gambia99_43 | 4 | 210 | 0 | 2.32428 | 0 |
| Gambia99_55 | 4 | 210 | 0 | 2.32428 | 0 |
| Gambia99_56 | 4 | 45 | 0 | 1.66276 | 0 |
| BurkinaDry_18 | 4 | 0 | 0 | 0 | 0 |
| BurkinaDry_22 | 4 | 2089 | 0 | 3.32015 | 0 |
| BurkinaDry_28 | 4 | 1072 | 24 | 3.0306 | 1.39794 |
| BurkinaDry_44 | 4 | 409 | 19 | 2.61278 | 1.30103 |
| BurkinaDry_61 | 4 | 1634 | 0 | 3.21352 | 0 |
| BurkinaDry_70 | 4 | 0 | 0 | 0 | 0 |
| BurkinaDry_76 | 4 | 0 | 0 | 0 | 0 |
| BurkinaDry_79 | 4 | 0 | 0 | 0 | 0 |
| BurkinaDry_81 | 4 | 0 | 0 | 0 | 0 |
| BurkinaDry_85 | 4 |  |  | 0 | 0 |
| BurkinaDry_88 | 4 | 0 | 0 | 0 | 0 |
| BurkinaWet_7 | 4 | 228 | 0 | 2.35984 | 0 |
| BurkinaWet_19 | 4 | 145 | 0 | 2.16435 | 0 |
| BurkinaWet_26 | 4 | 0 | 0 | 0 | 0 |
| BurkinaWet_32 | 4 | 0 | 0 | 0 | 0 |
| BurkinaWet_46 | 4 | 0 | 24 | 0 | 1.39794 |
| BurkinaWet_67 | 4 | 164 | 0 | 2.21748 | 0 |
| BurkinaWet_86 | 4 | 3960 | 158 | 3.5978 | 2.2014 |
| BurkinaWet_89 | 4 | 765 | 287 | 2.88423 | 2.45939 |
| BurkinaWet_92 | 4 | 5920 | 64 | 3.7724 | 1.81291 |
| BurkinaWet_97 | 4 | 7650 | 32 | 3.88372 | 1.51851 |
| BurkinaWet_101 | 4 | 14,462 | 16 | 4.16026 | 1.23045 |
| BurkinaWet_107 | 4 | 284 | 394 | 2.45484 | 2.5966 |
| BurkinaWet_114 | 4 | 39 | 105.5 | 1.60206 | 2.02735 |
| BurkinaWet_117 | 4 | 6846 | 48 | 3.8355 | 1.6902 |
| Gambia99_24 | 5 | 4 | 0 | 0.69897 | 0 |
| Gambia99_29 | 5 | 100 | 0 | 2.00432 | 0 |
| Gambia99_32 | 5 | 50 | 6 | 1.70757 | 0.845098 |
| Gambia99_34 | 5 | 15 | 7 | 1.20412 | 0.90309 |
| Gambia99_53 | 5 | 150 | 1 | 2.17898 | 0.30103 |
| BurkinaDry_10 | 5 | 0 | 0 | 0 | 0 |
| BurkinaDry_26 | 5 | 0 | 0 | 0 | 0 |
| BurkinaDry_37 | 5 | 4623 | 0 | 3.66502 | 0 |
| BurkinaDry_64 | 5 | 0 | 0 | 0 | 0 |
| BurkinaDry_68 | 5 | 370 | 0 | 2.56937 | 0 |
| BurkinaDry_71 | 5 | 0 | 0 | 0 | 0 |
| BurkinaDry_75 | 5 | 378 | 0 | 2.57864 | 0 |
| BurkinaDry_80 | 5 | 1105 | 0 | 3.04376 | 0 |
| BurkinaDry_93 | 5 | 4777 | 0 | 3.67925 | 0 |
| BurkinaDry_103 | 5 | 1280 | 0 | 3.10755 | 0 |
| BurkinaDry_105 | 5 | 1467 | 0 | 3.16673 | 0 |
| BurkinaDry_106 | 5 | 200 | 0 | 2.3032 | 0 |
| BurkinaWet_2 | 5 | 10,319 | 0 | 4.01368 | 0 |
| BurkinaWet_13 | 5 | 14,582 | 0 | 4.16385 | 0 |
| BurkinaWet_37 | 5 | 95 | 75 | 1.98227 | 1.88081 |
| BurkinaWet_54 | 5 | 8981 | 72 | 3.95337 | 1.86332 |
| BurkinaWet_60 | 5 | 39,238 | 0 | 4.59372 | 0 |
| BurkinaWet_64 | 5 | 666 | 0 | 2.82413 | 0 |
| BurkinaWet_65 | 5 | 0 | 0 | 0 | 0 |
| BurkinaWet_69 | 5 | 2090 | 0 | 3.32035 | 0 |
| BurkinaWet_73 | 5 | 402 | 19 | 2.6053 | 1.30103 |
| BurkinaWet_76 | 5 | 321 | 0 | 2.50786 | 0 |
| BurkinaWet_79 | 5 | 4601 | 269 | 3.66295 | 2.43136 |
| BurkinaWet_91 | 5 | 674 | 31 | 2.8293 | 1.50515 |
| BurkinaWet_94 | 5 | 0 | 16 | 0 | 1.23045 |
| BurkinaWet_115 | 5 | 5657 | 0 | 3.75266 | 0 |
| Gambia99_52 | 6 | 110 | 0 | 2.04532 | 0 |
| BurkinaDry_24 | 6 | 0 | 68 | 0 | 1.83885 |
| BurkinaDry_30 | 6 | 517 | 0 | 2.71433 | 0 |
| BurkinaDry_45 | 6 | 902 | 0 | 2.95569 | 0 |
| BurkinaDry_72 | 6 | 0 | 0 | 0 | 0 |
| BurkinaDry_78 | 6 | 1890 | 0 | 3.27669 | 0 |
| BurkinaWet_55 | 6 | 9874 | 0 | 3.99454 | 0 |
| BurkinaWet_56 | 6 | 1372 | 0 | 3.13767 | 0 |
| BurkinaWet_66 | 6 | 453 | 0 | 2.65706 | 0 |
| BurkinaWet_71 | 6 | 719 | 0 | 2.85733 | 0 |
| BurkinaWet_85 | 6 | 2182 | 0 | 3.33905 | 0 |
| BurkinaWet_88 | 6 | 1621 | 62 | 3.21005 | 1.79934 |
| BurkinaWet_106 | 6 | 10,117 | 156 | 4.00509 | 2.1959 |
| BurkinaDry_2 | 7 | 452 | 0 | 2.6561 | 0 |
| BurkinaDry_9 | 7 | 0 | 12 | 0 | 1.11394 |
| BurkinaDry_19 | 7 | 1652 | 0 | 3.21827 | 0 |
| BurkinaDry_49 | 7 | 562 | 0 | 2.75051 | 0 |
| BurkinaDry_62 | 7 | 925 | 0 | 2.96661 | 0 |
| BurkinaWet_9 | 7 | 955 | 0 | 2.98046 | 0 |
| BurkinaWet_39 | 7 | 6891 | 12 | 3.83835 | 1.11394 |
| BurkinaWet_43 | 7 | 497 | 0 | 2.69723 | 0 |
| BurkinaWet_44 | 7 | 1037 | 0 | 3.0162 | 0 |
| BurkinaWet_45 | 7 | 5846 | 0 | 3.76693 | 0 |
| BurkinaWet_48 | 7 | 350 | 0 | 2.54531 | 0 |
| BurkinaWet_51 | 7 | 354 | 8 | 2.55023 | 0.954243 |
| BurkinaWet_57 | 7 | 0 | 0 | 0 | 0 |
| BurkinaWet_77 | 7 | 71 | 0 | 1.85733 | 0 |
| BurkinaDry_6 | 8 | 0 | 0 | 0 | 0 |
| BurkinaDry_13 | 8 | 5406 | 0 | 3.73296 | 0 |
| BurkinaDry_21 | 8 | 160 | 0 | 2.20683 | 0 |
| BurkinaDry_29 | 8 | 637 | 0 | 2.80482 | 0 |
| BurkinaDry_38 | 8 | 2759 | 32 | 3.44091 | 1.51851 |
| BurkinaDry_63 | 8 | 0 | 0 | 0 | 0 |
| BurkinaDry_66 | 8 | 0 | 0 | 0 | 0 |
| BurkinaDry_69 | 8 | 20 | 0 | 1.32222 | 0 |
| BurkinaDry_87 | 8 | 1259 | 0 | 3.10037 | 0 |
| BurkinaDry_97 | 8 | 872 | 20 | 2.94101 | 1.32222 |
| BurkinaDry_99 | 8 | 380 | 0 | 2.58092 | 0 |
| BurkinaWet_16 | 8 | 108 | 0 | 2.03743 | 0 |
| BurkinaWet_83 | 8 | 14,679 | 572 | 4.16673 | 2.75815 |
| BurkinaWet_110 | 8 | 5707 | 16 | 3.75648 | 1.23045 |
| BurkinaWet_116 | 8 | 1477.5 | 34 | 3.16982 | 1.54407 |
| BurkinaDry_5 | 9 | 0 | 0 | 0 | 0 |
| BurkinaDry_12 | 9 | 361 | 0 | 2.55871 | 0 |
| BurkinaDry_17 | 9 | 0 | 0 | 0 | 0 |
| BurkinaDry_25 | 9 | 111 | 8 | 2.04922 | 0.954243 |
| BurkinaDry_98 | 9 | 0 | 0 | 0 | 0 |
| BurkinaWet_24 | 9 | 336 | 0 | 2.52763 | 0 |
| BurkinaWet_40 | 9 | 528 | 0 | 2.72346 | 0 |
| BurkinaWet_42 | 9 | 4730 | 0 | 3.67495 | 0 |
| BurkinaWet_52 | 9 | 390 | 0 | 2.59218 | 0 |
| BurkinaWet_61 | 9 | 116 | 8 | 2.06819 | 0.954243 |
| BurkinaWet_74 | 9 | 155 | 23 | 2.19312 | 1.38021 |
| BurkinaWet_95 | 9 | 6030 | 16 | 3.78039 | 1.23045 |
| BurkinaWet_109 | 9 | 1193 | 32 | 3.077 | 1.51851 |
| BurkinaDry_33 | 10 | 39 | 0 | 1.60206 | 0 |
| BurkinaDry_52 | 10 | 2125 | 0 | 3.32756 | 0 |
| BurkinaDry_53 | 10 | 1727 | 0 | 3.23754 | 0 |
| BurkinaDry_83 | 10 | 0 | 0 | 0 | 0 |
| BurkinaDry_104 | 10 | 1557 | 0 | 3.19257 | 0 |
| BurkinaWet_3 | 10 | 879 | 8 | 2.94448 | 0.954243 |
| BurkinaWet_14 | 10 | 1570 | 0 | 3.19618 | 0 |
| BurkinaWet_15 | 10 | 389 | 0 | 2.59106 | 0 |
| BurkinaWet_84 | 10 | 2927 | 16 | 3.46657 | 1.23045 |
| BurkinaDry_27 | 11 | 0 | 0 | 0 | 0 |
| BurkinaDry_34 | 11 | 0 | 0 | 0 | 0 |
| BurkinaDry_43 | 11 | 0 | 0 | 0 | 0 |
| BurkinaDry_50 | 11 | 1377 | 577 | 3.13925 | 2.76193 |
| BurkinaDry_51 | 11 | 1825 | 0 | 3.2615 | 0 |
| BurkinaDry_59 | 11 | 743 | 0 | 2.87157 | 0 |
| BurkinaDry_100 | 11 | 6339 | 0 | 3.80209 | 0 |
| BurkinaWet_82 | 11 | 25,986 | 56 | 4.41476 | 1.75587 |
| BurkinaDry_58 | 12 | 827 | 0 | 2.91803 | 0 |
| BurkinaDry_73 | 12 | 122 | 0 | 2.08991 | 0 |
| BurkinaDry_82 | 12 | 0 | 0 | 0 | 0 |
| BurkinaWet_30 | 12 | 13,022 | 8 | 4.11471 | 0.954243 |
| BurkinaWet_31 | 12 | 2869 | 8 | 3.45788 | 0.954243 |
| BurkinaWet_53 | 12 | 534 | 0 | 2.72835 | 0 |
| BurkinaDry_31 | 13 | 425 | 64 | 2.62941 | 1.81291 |
| BurkinaWet_4 | 13 | 2992 | 0 | 3.47611 | 0 |
| BurkinaWet_23 | 13 | 652 | 0 | 2.81491 | 0 |
| BurkinaWet_38 | 13 | 817 | 0 | 2.91275 | 0 |
| BurkinaDry_41 | 14 | 3744 | 0 | 3.57345 | 0 |
| BurkinaDry_60 | 14 | 0 | 0 | 0 | 0 |
| BurkinaWet_6 | 15 | 855 | 0 | 2.93247 | 0 |
| BurkinaWet_12 | 16 | 1552 | 0 | 3.19117 | 0 |
| BurkinaWet_50 | 17 | 778 | 0 | 2.89154 | 0 |
| BurkinaWet_28 | 18 | 2605 | 4 | 3.41597 | 0.69897 |
