# Supplementary material for: Transmission of molecularly undetectable circulating parasite clones leads to high infection complexity in mosquitoes post feeding
Source: Int J Parasitol. 2018 Jul;48(8):671–7. doi: 10.1016/j.ijpara.2018.02.005 (PMC6018601; doi:10.1016/j.ijpara.2018.02.005)
Supplement: Supplementary data 3 — Supplementary Fig. S1. Plasmodium falciparum alleles unique in mosquitoes and matched in mosquitoes by allele size. (A) Proportion of unique mosquito alleles and alleles matched in mosquitoes and the human blood they had fed on, by allele size and allelic family (3D7 allelic family). (B) Proportion of unique mosquito alleles and alleles matched in mosquitoes and the human blood they had fed on, by allele size and allelic family (Fc27 allelic family). Supplementary Fig. S2. Number of Plasmodium falciparum clones transmitted to mosquitoes that remained undetected in the human blood on which the mosquitoes fed, by asexual parasite density (A) and gametocyte density (B). Supplementary Fig. S3. Unique Plasmodium falciparum alleles in mosquitoes per feeding assay by number of mosquitoes analysed. [file mmc3.docx]

Supplementary Fig. S1

| A |
| --- |
|  |

| B |
| --- |
|  |

Supplementary Fig. S2

| A | B |
| --- | --- |
|  |  |

|  |
| --- |

Supplementary Fig. S3
